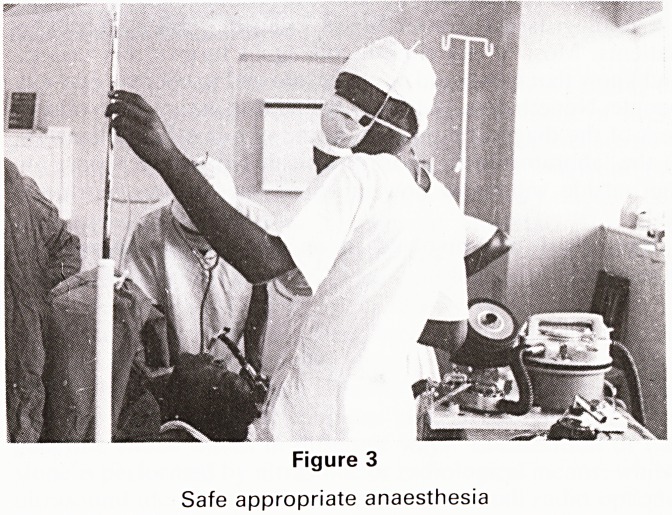# From Our Foreign Correspndents

**Published:** 1988-08

**Authors:** 


### From Our Foreign Correspondents

### Surgical Safari

Wandering alone at dawn along the shore of the Jade Sea (Lake Turkana) in northern Kenya I feel yet again the smallness that comes from being in the vastness that is Africa. The mountains, Rift valley, lakes, plains and over all the wonderful skies are almost overwhelming. My surgical journeying really began with a working trip to a small mission hospital at Raxaul on the north Indian border with Nepal. The toughess and fortitude especially of the Nepalis who came to us over the border were impressive. A bonus on my weekend off was a 15-minute flight over the Himalayas to Kathmandu where I visited the famous Shanti Bahwan hospital.

But perhaps the love affair with Africa started with a spell as RAMC surgeon in Cyrenaica (now Libya) just after World War II and has burst into flame again in recent years during several surgical locums in Kenya and Zambia. So that same sense of smallness comes as I see the never-ending crowds of people needing surgical care. Hopefully 'every little helps' but a surgeon soon realises he is dealing with problems better prevented than cured, such as appalling burns in children. In 'community medicine' lies the best hope for many of the ills of Africa, and immunization, nutrition, family planning, antenatal care and health education hardly come within the surgeon's usual scope. Yet in the absence of primary care as we know it, the rural hospital with its tradition and trusted practice of curative medicine and safe surgery and maternity, forms the best focal point for such a programme, combined with training of nurses, field workers and traditional birth attendants.

**Figure 1**

Burns overshadow all other injuries

The Presbyterian Hospital at Chogoria, at 5000 feet on the slopes of Mount Kenya, is such a place. Serving a population of some 300,000 (similar to the Royal Devon and Exeter), it has 300 beds, 34 district clinics, and does some 1000 major operations a year. With an excellent maternity service, a family planning service which over the years has reduced the local fertility rate to 4.7 children per family as opposed to an average of 7.3 in all Kenya, goes a high child immunisation rate (though measles is still a killer), but enough drunken driving to produce lethal trauma. For a 'general surgeon with vascular experience' African trauma is quite exciting. Depressed skull fracture, stab wounds of chest or abdomen, neglected compound fracture of limbs, multiple pile-ups as well as snake, buffalo or crocodile injuries are all overshadowed by burns (Fig 1), especially childrens burns. In an anaemic, mal-nourished child, cleaning up and really early split-skin grafting can be life-saving.

**Figure 2**

Poster warning of AIDS risk

Sepsis in all its most bizarre forms includes recurrent osteomyelitis with sequestration, multiple pyomyositis with abscesses, and gross pelvic inflammatory disease. When the microbiologist is missing and antibiotics are limited, the surgical precepts of adequate drainage, deroofing and antiseptic dressings are still of proven value.

Yet in some ways rural African surgical care runs ahead of Western ways. Blood transfusion is used sparingly and only to save life, autotransfusion is normal practice in ruptured ectopic pregnancy and certain trauma, and blood donors usually come from the family. Antimalarial treatment is usually given with the blood, but recognition of the risks of hepatitis and AIDS is dawning, (Fig 2) and in some areas where screening of donors has been done the results are disquieting.

But how, you may say, can *safe* surgery be practised under such limitations? In each place where I have had the good fortune to work, well-trained theatre staff, good aseptic technique, safe appropriate anaesthesia (Fig 3) and meticulous aftercare have resulted in amazingly low morbidity and mortality rates, discussed at regular staff meetings. Thyroidec-

**Figure 3**

Safe appropriate anaesthesia tomy using draw-over ether (no diathermy) after intubation by an adept clinical officer, prostatectomy under spinal, children's burns and other operations under Ketalar, and judicious use of local and regional blocks, pose only easily surmountable problems.

It has been said that a surgeon needs 'availability, affability and ability' in that order. For work in rural Africa we might add 'adaptability and appropriateness' based on the widest and most general experience one can obtain, something sadly increasingly difficult under our present rather rigid system with early specialisation. Though the concept of 'team work' in surgery is important, it is nowhere more so than in rural Africa that the surgeon has to 'make all the running' and the continual need to 'press on' can be demanding.

Apart from the surgical care of needy patients, the most useful work is appropriate training in safe 'low-tech' surgery. History taking is difficult, not only because of language problems, so there must be reliance on carefully elicited physical signs, judgement so that the inappropriate and impossible are not attempted, and detailed and meticulous postoperative orders and supervision.

Cancer is a different problem in Africa. Two of our accepted arms of treatment, radiotherapy and chemotherapy, are often not possible, so that surgery may be the only choice, to be undertaken only for cure (as in amputation for squamous-cell carcinoma in a long-standing leg ulcer), or for real palliation (as in gastroenterostomy in a patient with pyloric obstruction.) It is humbling to see how most Africans face the truth about a serious prognosis in a brave and phlegmatic way, though nowadays the accepted support of the 'extended family' is not always forthcoming and the relatives disappear. Research is by no means impossible, and useful studies continue to be made and published by enthusiasts.

After five surgical safaris, I now have great respect for African patients, nurses, paramedics and doctors, less for administrators. A hospital of a size to be run, as in times past, by a threesome of doctor, matron and secretary-manager can have its staff, finance, facilities, drugs and community outreach well organised. But a 2000-bed teaching hospital trying to emulate its European or American counterpart is too big to manage, and reaches only nearby patients with its often badly maintained high-tech service.

This was only too apparent at the University Teaching Hospital in Lusaka, where operating lists were cancelled because of 'no blood' (simply because the blood van had broken down), because of 'no atropine' (because of failure to order), or because of 'no gloves' (because in face of a shortage they refused to learn how to repack and resterilize as we always did in WW II). The devoted mainly expatriate surgical staff struggle on with excellent teaching and research. So why are 80% of Kenya's doctors in Nairobi and Mombasa? Unfortunately the teaching and practice on safe, low-tech surgery carries little kudos. Support is needed for the growing awareness that training must meet this challenge. The Royal College's Overseas Doctors' Training Scheme in Surgery needs our support, with the emphsis on supervised experience, safe bloodless craftmanship, good judgement, less emphasis on radiology more on endoscopy and laparoscopy. The supervision and help for these trainees will be a real challenge and vocation for some of us.

So what's surgical safari worth? With the retrospectoscope, hopefully, some bodies mended, some suffering lessened, much thankfulness for one's training and trainers, confirmation of one's faith in up-and-coming keen young doctors and nurses, a recognition that time spent working abroad is a valuable part of the training of a competent surgeon. So when are we off again?

KEITH VOWLES
Exeter

### Anastomosis Workshop in Saudi Arabia

The anastomosis workshop begun in the early 1980's by Professor Peter Bevan at the Royal College of Surgeons of England continue to flourish and to expand to other institutions, and to other countries. I have been a demonstrator to the workshop both at the Buckston-Browne farm in Kent and latterly once a year at the College itself in London. The workshops attract 20 surgeons-in-training at each session who are shown gastrointestinal, vascular, urological and stapled anastomoses using fresh porcine bowel, blood vessels and ureters on specially designed jigs. My brief has been stapled gastrointestinal and hand sewn colorectal diseases.

Following a successful visit to Kuwait two years ago a group of us were invited to run a similar workshop at the King Fahad Hospital—a National Guard hospital in Riyadh, Saudi Arabia. Professor Peter Bevan (Birmingham), Mr Jerry Kirk (London), Mr Felix Eastcott (London), Mr Peter Lee (Hull), Mr Peter Thompson (London), Mr Ian Capperauld (Edinburgh) and I ran a four day workshop with tutorials in the usual way. The twenty attending Saudi surgeons-in-training were as responsive and as good as their UK counterparts. Interestingly a final FRCS session recently held there passed 4 of 5 women and 5 of 25 men surgeons.

We were treated to a brief look at Saudi medicine and surgery on ward rounds on our final day. Like everything in this country there was no shortage of equipment with a liberal importation of expertise from both local Arabic countries and the so called West. One of our hosting surgeons dealt with three cases of liver trauma whilst we were there—all appeared to be making good progress as we were leaving.

My other memories of this country will be the remarkable building and roadworks programme. Riyadh is a young city in terms of its development and interspersed with the new are the mud and straw buildings of the past which were lived in until quite recently in Diriyah. The new football stadium and University are the most impressive additions to the skyline.

Other memories . . . . oh yes . . . . . playing golf on "browns" at the Riyadh Desert Golf Club . . . . a large gin and tonic on the Boeing 747—sorry sir this is Saudia Airlines—we're dry.

D. J. LEAPER
Bristol

## Figures and Tables

**Figure 1 f1:**
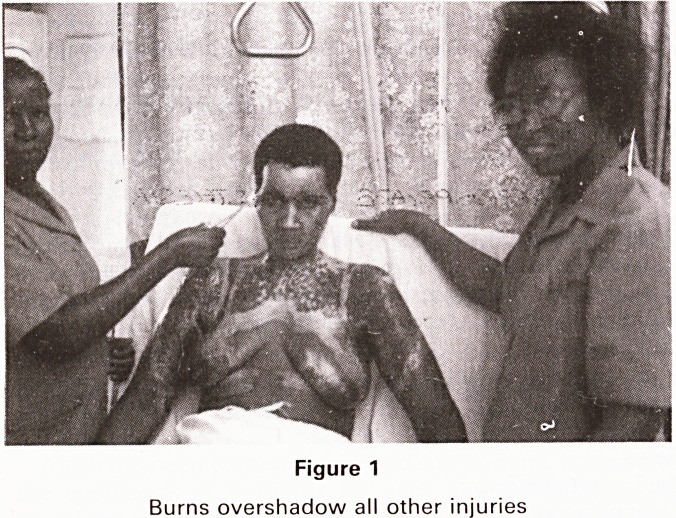


**Figure 2 f2:**
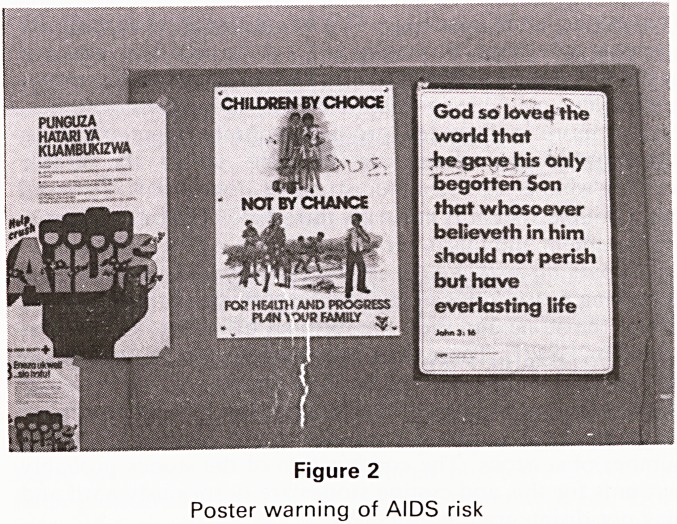


**Figure 3 f3:**